# Supplementary material for: Pressure Point Thresholds and ME/CFS Comorbidity as Indicators of Patient’s Response to Manual Physiotherapy in Fibromyalgia
Source: Int J Environ Res Public Health. 2020 Oct 31;17(21):8044. doi: 10.3390/ijerph17218044 (PMC7662886; doi:10.3390/ijerph17218044)
Supplement: Supplementary file 1 [file ijerph-17-08044-s001.zip › Supplementary Files-Proofs/Supplementary Figure S1.pdf]

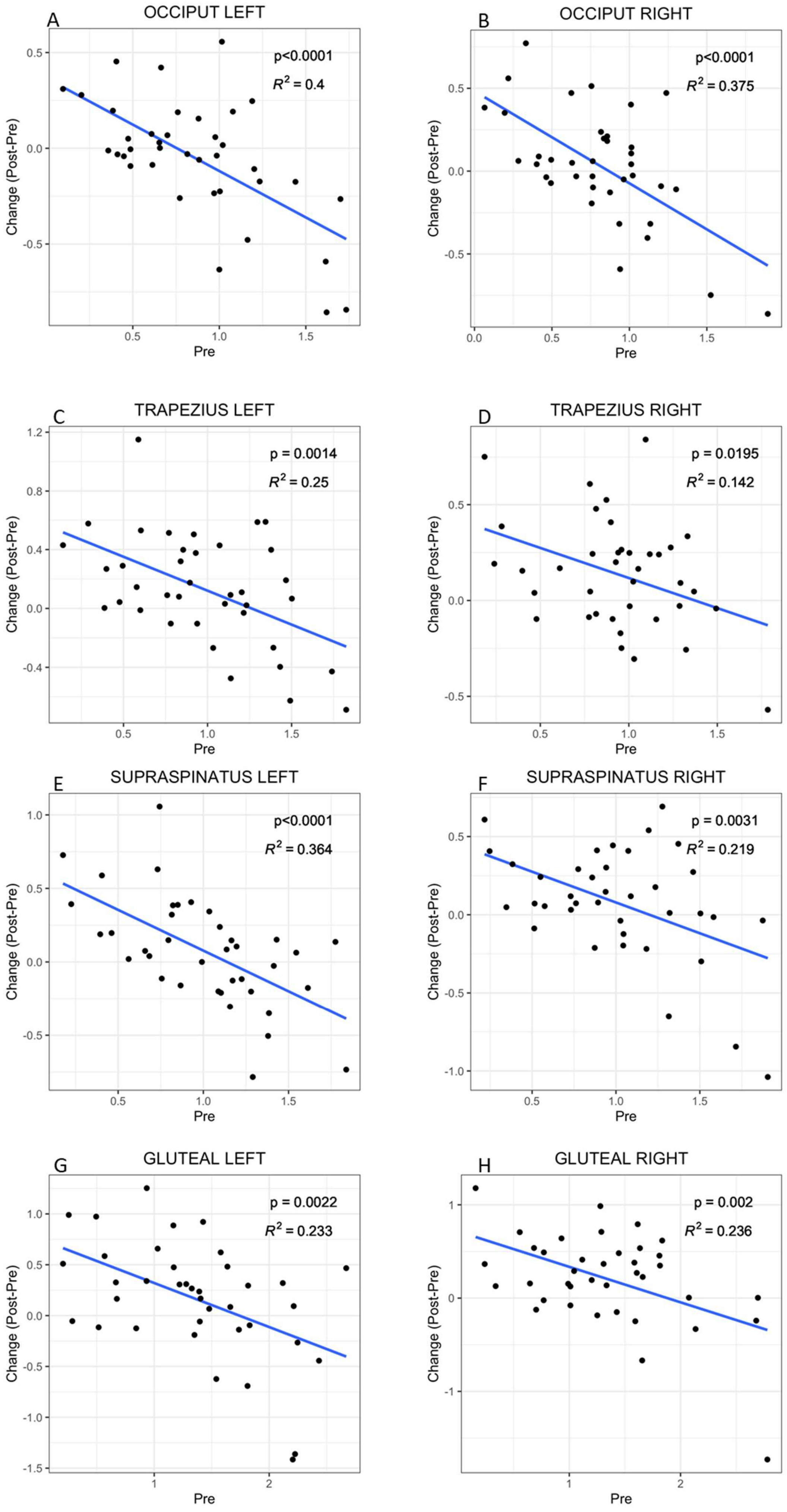
A
OCCIPUT LEFT
p<0.0001
R^2 = 0.4
B
OCCIPUT RIGHT
p<0.0001
R^2 = 0.375
C
TRAPEZIUS LEFT
p = 0.0014
R^2 = 0.25
D
TRAPEZIUS RIGHT
p = 0.0195
R^2 = 0.142
E
SUPRASPINATUS LEFT
p<0.0001
R^2 = 0.364
F
SUPRASPINATUS RIGHT
p = 0.0031
R^2 = 0.219
G
GLUTEAL LEFT
p = 0.0022
R^2 = 0.233
H
GLUTEAL RIGHT
p = 0.002
R^2 = 0.236
Change (Post-Pre)
Pre

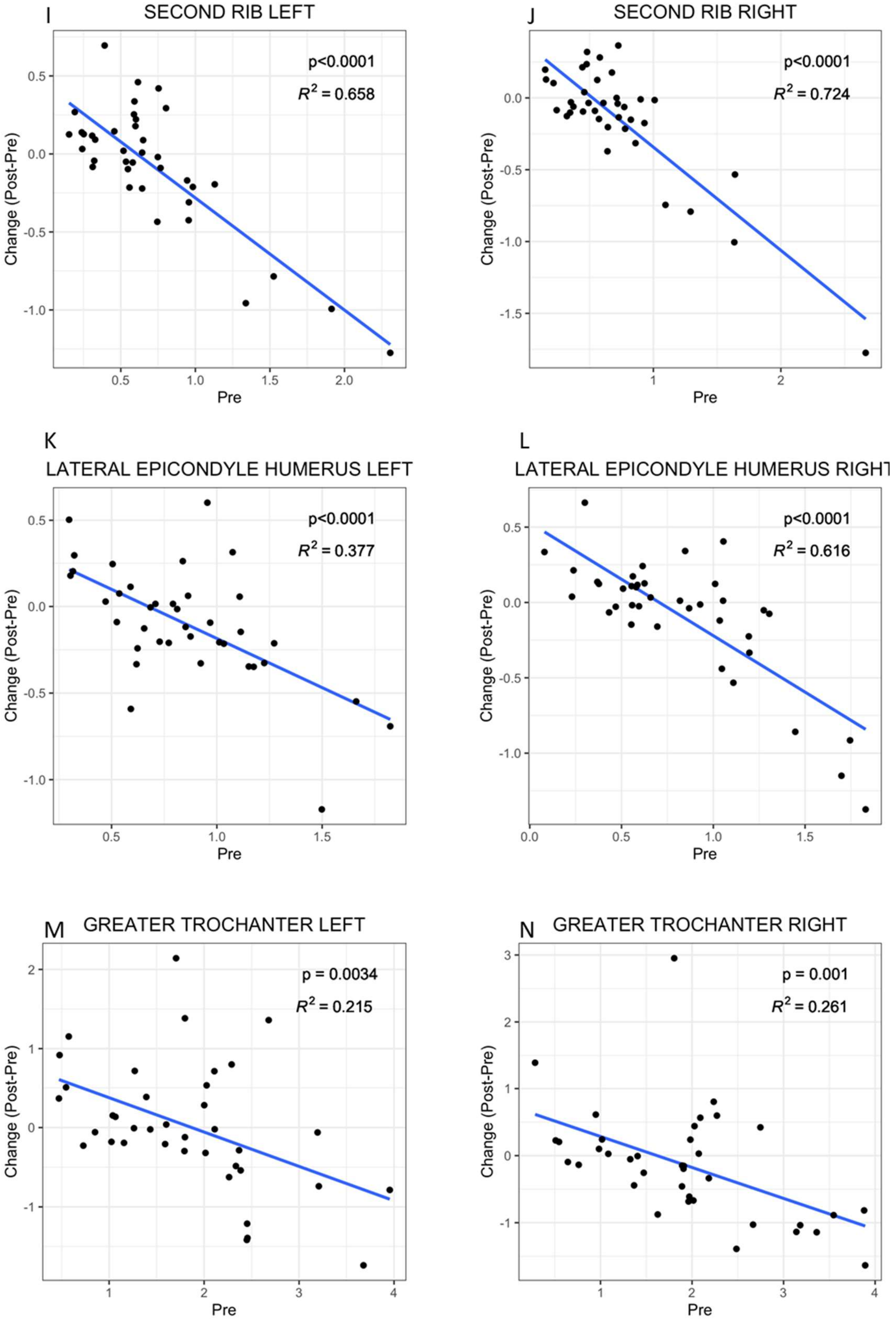

Supplementary Figure S1. Linear dependence of PPT baseline scores with MT therapy response. The plots show the inverse correlation between pre-treatment PPT values and the improvement measured as the acquired resistance to pressure-induced pain for the tender points (Change: Post-Pre) at the indicated tender points (A-N). P and R2 values are shown.
