# Supplementary material for: Pressure Point Thresholds and ME/CFS Comorbidity as Indicators of Patient’s Response to Manual Physiotherapy in Fibromyalgia
Source: Int J Environ Res Public Health. 2020 Oct 31;17(21):8044. doi: 10.3390/ijerph17218044 (PMC7662886; doi:10.3390/ijerph17218044)
Supplement: Supplementary file 1 [file ijerph-17-08044-s001.zip › Supplementary Files-Proofs/Supplementary Figure S2.pdf]

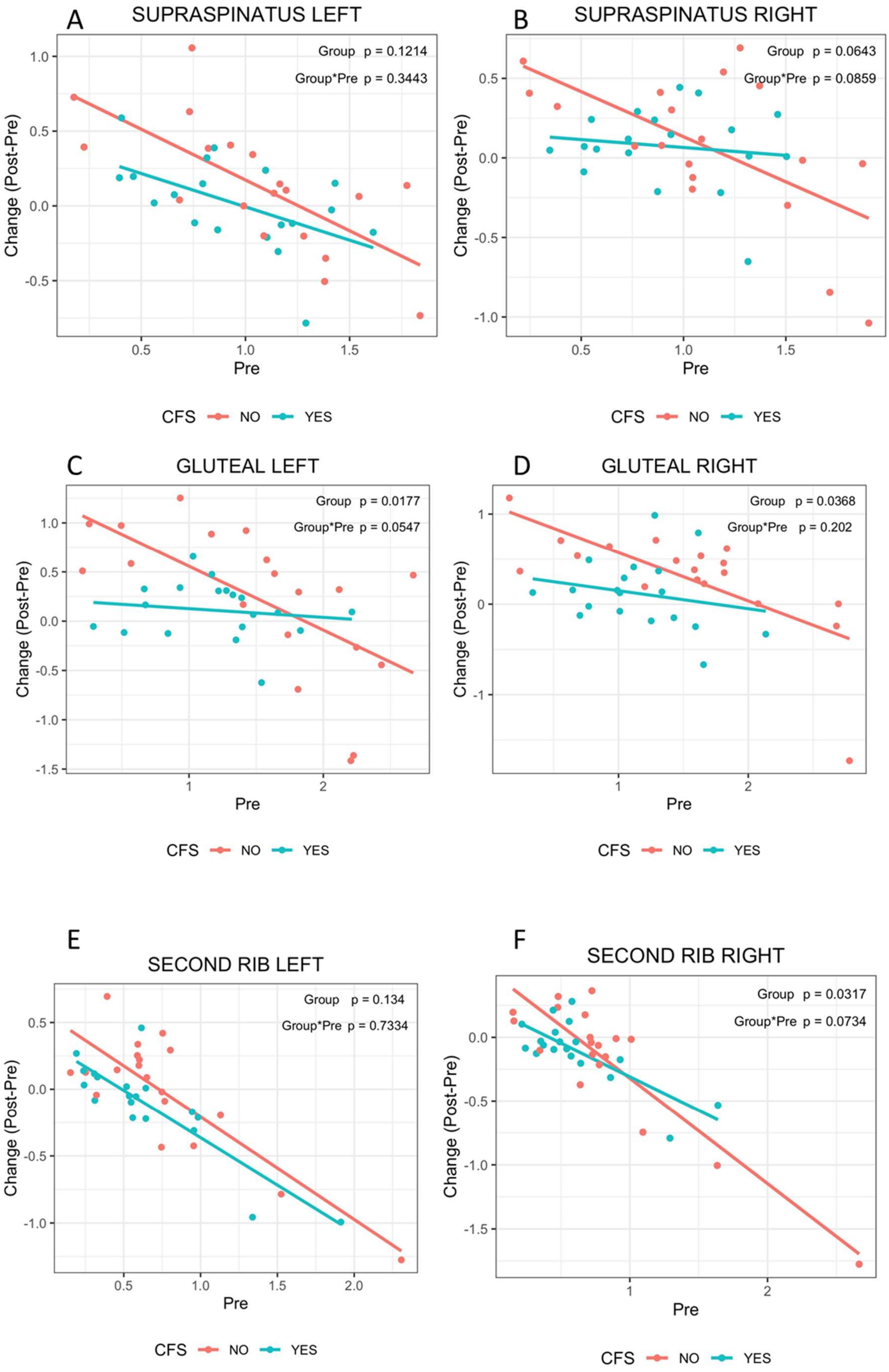

A
SUPRASPINATUS LEFT
Group p = 0.1214
Group*Pre p = 0.3443
Change (Post-Pre)
1.0
0.5
0.0
-0.5
0.5
1.0
1.5
Pre
CFS NO YES
B
SUPRASPINATUS RIGHT
Group p = 0.0643
Group*Pre p = 0.0859
Change (Post-Pre)
0.5
0.0
-0.5
-1.0
0.5
1.0
1.5
Pre
CFS NO YES
C
GLUTEAL LEFT
Group p = 0.0177
Group*Pre p = 0.0547
Change (Post-Pre)
1.0
0.5
0.0
-0.5
-1.0
-1.5
1
2
Pre
CFS NO YES
D
GLUTEAL RIGHT
Group p = 0.0368
Group*Pre p = 0.202
Change (Post-Pre)
1
0
-1
1
2
Pre
CFS NO YES
E
SECOND RIB LEFT
Group p = 0.134
Group*Pre p = 0.7334
Change (Post-Pre)
0.5
0.0
-0.5
-1.0
0.5
1.0
1.5
2.0
Pre
CFS NO YES
F
SECOND RIB RIGHT
Group p = 0.0317
Group*Pre p = 0.0734
Change (Post-Pre)
0.0
-0.5
-1.0
-1.5
1
2
Pre
CFS NO YES

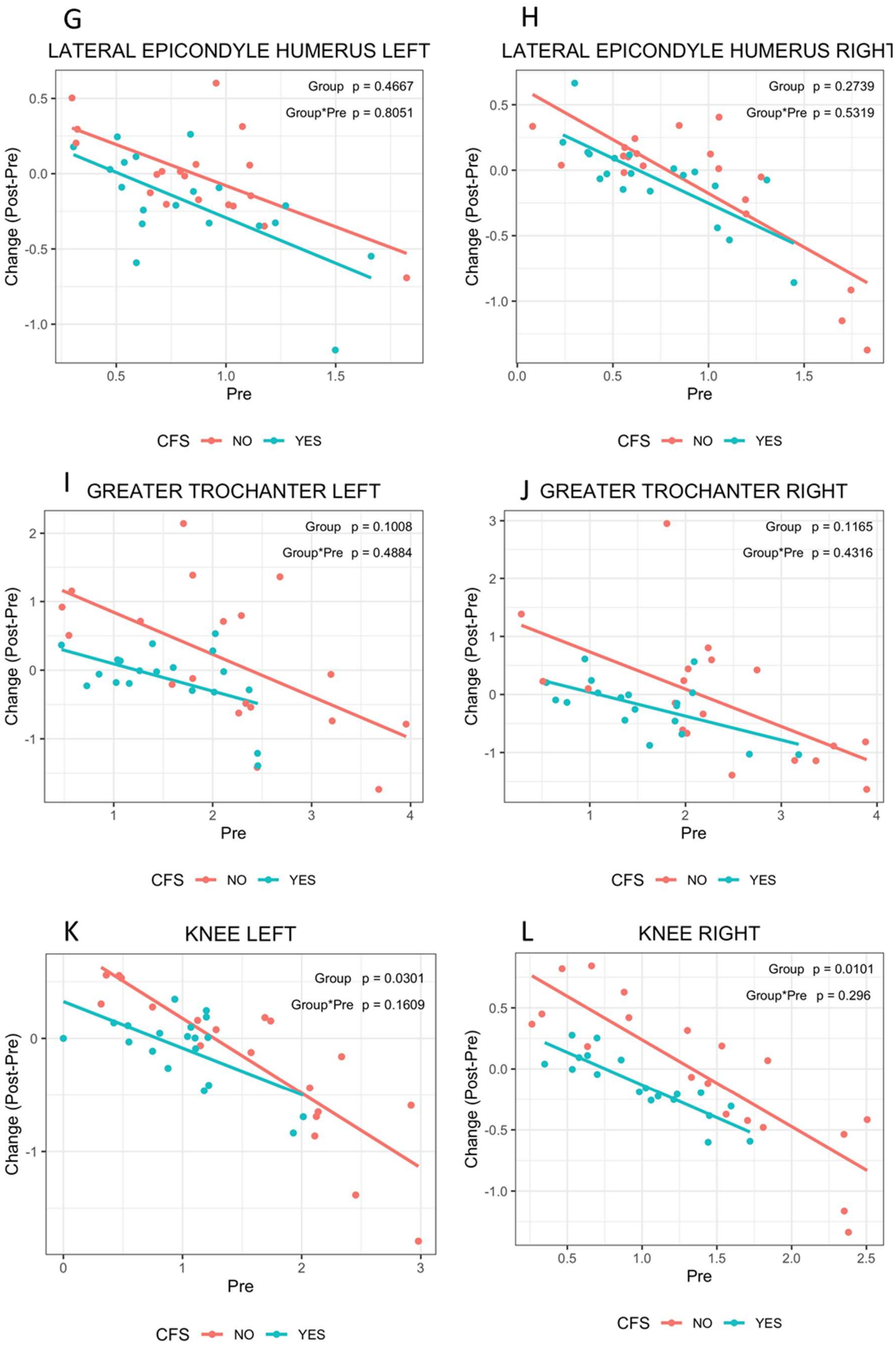

Supplementary Figure S2. Linear dependence of PPT baseline scores (Pre) with MT therapy response (Change: Post-Pre) in patients presenting FM with or without comorbid ME/CFS. The plots show the inverse correlation between pre-treatment PPT values and symptom improvement, measured as the acquired resistance to pressure-induced pain for the tender points indicated (A-L). Linear adjustments in the FM only subgroup are shown in red color, while those for the FM patients that present with ME/CFS comorbidity are shown in green. P values of the differences between FM only and FM with ME/CFS are shown.
